# A population-based analysis of use and outcomes of laparoscopic bariatric surgery across socioeconomic groups in Taiwan

**DOI:** 10.1186/s12939-015-0265-9

**Published:** 2015-11-11

**Authors:** Chun-Che Huang, Yu-Tung Huang, Chong-Chi Chiu

**Affiliations:** Institute of Health Policy and Management, National Taiwan University, Taipei, Taiwan; Master Degree Program in Aging and Long-Term Care, College of Nursing, Kaohsiung Medical University, 100, Shih-Chuan 1st Road, Kaohsiung, Taiwan; Chronic Diseases and Health Promotion Research Center, Chang Gung University of Science and Technology, Chiayi, Taiwan; Department of General Surgery, Chi Mei Medical Center, Tainan, Liouying Taiwan; Department of Electrical Engineering, Southern Taiwan University of Science and Technology, Tainan, Taiwan

**Keywords:** Socioeconomic status, Morbid obesity, Laparoscopic bariatric surgery

## Abstract

**Background:**

With the growing development of minimally invasive techniques for the treatment of morbid obesity, laparoscopic bariatric surgery (LBS) is increasingly performed. This study aimed to assess the association between patients’ socioeconomic status (SES) and the likelihood of undergoing LBS and related outcomes in Taiwan.

**Methods:**

This nationwide population-based study was conducted by using data from Taiwan’s National Health Insurance Research Database. A total of 3678 morbidly obese patients aged 18 years and older who underwent conventional open bariatric surgery or LBS were identified between 2004 and 2011. Regression analyses were performed using generalized estimating equation (GEE) models to account for the nesting of patients within physician to assess patients’ SES category associated with the use of LBS and related outcomes. Odds ratios (ORs) and 95 % confidence intervals (CIs) were estimated.

**Results:**

Compared with those with medium and low SES (84.6 % and 80.2 %), patients with high SES (88.1 %) had the highest percentage of undergoing LBS (*P* < 0.001). After adjusting for patient demographics, institution and surgeon characteristics, the multivariate GEE analysis revealed that the highest likelihood of undergoing LBS was noted in morbidly obese patients with high SES (OR = 1.45, 95 % CI 1.10–1.90), followed by those with medium SES (OR = 1.27, 95 % CI 1.04–1.56). In addition, patients with high SES had slightly lower length of hospital stay (LOS; OR = 0.90, 95 % CI 0.82–0.99) and hospital treatment cost (OR = 0.93, 95 % CI 0.87–0.99) than their counterparts after adjustment.

**Conclusions:**

The increased likelihood of undergoing LBS and lower LOS and hospital treatment cost were noted among morbidly obese patients with higher SES. This finding suggests there is the need to improve clinical practice and reduce health disparities in the surgical treatment of morbidly obese patients.

## Background

Bariatric surgery has been proven as an attractive and effective control method for morbid obesity and associated metabolic disorders [1, 2]. With the development of advanced surgical instrumentation and introduction, the numbers of laparoscopic bariatric procedures have grown substantially in developed and developing countries in the past decade [3–5]. In Taiwan, the annual number of bariatric surgery cases, particularly laparoscopic bariatric surgery (LBS), has increased over the past few years [6].

However, LBS is only covered by Taiwan’s National Health Insurance (NHI) payment for surgical fee, ward fee, examination fee, treatment fee and medication fee, not for some of special material fees of LBS. The average surgical costs for LBS per patient had to pay extra-expenses between 150,000 and 250,000 new Taiwan dollars (approximately 4688–7813 US dollars) [3]. In addition, hospital treatment costs at different hospital levels were reimbursed under the national health insurance system, the highest of which were generally received by medical centers and the lowest of which were received by local hospitals [6].

Numerous studies have shown that gastric surgery with laparoscopic assistance has the advantages of low morbidity, short length of hospital stay (LOS), expedient return to normal activity [1, 7], and improved patients’ quality of life [8]. However, this procedure also carries a potential risk of perioperative complications [2, 7] and has a recognized steep learning curve, even for experienced surgeons [3, 5, 9]. To our knowledge, although the use of bariatric surgery differs considerably across socioeconomic groups [10–13], it is unclear whether the choice of LBS could be influenced by socioeconomic status (SES). In addition, previous studies focused on the eligibility of patients for bariatric procedures across socioeconomic groups based on regional samples and less relevant to adjustment. This study thus used a population-based dataset to evaluate whether morbidly obese patients with high SES are more likely to undergo LBS and related outcomes under the national health insurance coverage system.

## Methods

### Data sources

This retrospective population-based cross-sectional study retrieved data from the Taiwan’s National Health Insurance Research Database (NHIRD) between 2004 and 2011, including the inpatient expenditures by admissions, registry for contracted medical facilities, registry for medical personnel and registry for beneficiaries. All datasets for the relevant variables were linked using the scrambled unique personal or medical institutional identification number which was encrypted by the National Health Research Institutes (NHRI). The study strictly adhered to the regulations regarding data privacy and confidentiality protection. The procedure and diagnosis codes for each hospitalization were categorized on the basis of the International Classification of Diseases, Ninth Revision, Clinical Modification (ICD-9-CM) coding. The requirements for standardized procedures were mandated by the National Health Insurance Administration (NHIA) and performed a quarterly expert review to ensure quality of care and accuracy of the claim files. In addition, this study was approved by the Institutional Review Board of the Chang Gung Memorial Hospital (102–1378B, Taoyuan, Taiwan).

### Study population

A total of 3713 patients aged 18 years or older with the principal diagnosis code for overweight or obesity (ICD-9-CM codes 278.00–278.02 or 278.1) are eligible for bariatric surgery according to Taiwan’s NHIA reimbursement policy [6]. Primary procedure code for bariatric operations, including open gastric bypass surgery (44.31 and 44.39), laparoscopic gastric bypass surgery (44.38), open gastroplasty (44.69), laparoscopic gastroplasty (including laparoscopic vertical banded gastroplasty, 44.68 and laparoscopic adjustable gastric band, 44.95) and sleeve gastrectomy (43.89) [6]. We excluded 35 cases (0.9 %) had diagnosis codes for gastrointestinal tract neoplasm (150.0–159.9), inflammatory bowel disease (555.0–556.9) or noninfectious colitis (557.0–558.9), and those underwent emergent procedures [1]. Our final sample included 3678 patients who underwent conventional open or laparoscopic-assisted bariatric surgeries.

### Outcome measures

The primary outcome measure was whether or not a patient had bariatric surgery with laparoscopy. Laparoscopic bariatric surgery (LBS) with different level of laparoscopic assistance was included laparoscopic mini-gastric bypass, laparoscopic Roux-en-Y gastric bypass and laparoscopic vertical banded gastroplasty. However, because the new ICD-9-CM laparoscopic bariatric surgical codes which was not readily used in practice until 2006 were infrequent in Taiwan, we combined with a procedure modifier code for laparoscopy (54.21) were identified as patients who had underwent LBS [14]. In addition, secondary outcome measures were the occurrence of surgical complications, length of the hospital stay, and hospital treatment cost. The procedure related complications for bariatric surgery were defined by ICD-9-CM diagnosis codes, including complications peculiar to certain specific procedures (996.x), complications affecting specific body systems not elsewhere specified (997.x), and other complications of procedures (998.x) [15].

### Main exposure and covariates

The primary independent variable was patients’ socioeconomic status (SES), which was defined as beneficiaries’ insurable monthly wages from the NHIRD registry for beneficiaries. Since the Taiwan’s National Health Insurance (NHI) scheme is financed by wage-based premiums for people with a clearly defined insurable monthly wage and by fixed premiums for people without a defined insurable monthly wage. SES was divided into three categories: low (< NT$20,000), medium (NT$20,000–39,999) and high (≥ NT$40,000). Additionally, patients without a clearly defined insurable monthly wage were mostly vulnerable people, such as farmers, fishermen and low-income people, and they were assigned to the same low socioeconomic status group as those people with insurable wage less than NT$20,000.

The covariates were selected according to the literature review and the information available in the database, were as follows: characteristics of patients (including age, gender, beneficiaries’ geographic location, comorbidities and year of operation), hospitals (including accreditation level) and surgeons (including age and surgical volume).

We obtained information on the beneficiaries’ location of residence or workplace from the NHIRD registry for beneficiaries. The region of each patient’s NHIA unit was divided into four regions (Northern, Central, Southern and Eastern) according to the National Statistics of Regional Standard Classification. The inpatient expenditures by admissions provided information on patient characteristics. We used the Deyo modification of the Charlson Comorbidity Index (CCI) score to adjust case mix for severity of comorbid illness with administrative data [16]. Additionally, patients with obesity-related comorbidities were categorized as follows: diabetes mellitus (ICD-9-CM code 250), dyslipidemia (272.0, 272.1, 272.2, 272.3 and 272.4), hypertension (401–405) and obstructive sleep apnea (OSA; 780.51, 780.53 and 780.57) [17, 18].

Information on characteristics of hospitals and surgeons was retrieved from the registry for contracted medical facilities and medical personnel. Hospital accreditation level was classified into medical centers, regional and district hospitals based on the Taiwan’s hospital accreditation system. Surgical volume was determined based on the average annual number of bariatric operations performed by each surgeon during 2004–2011 and was classified into low (<15 patients/year) and high (≥15 patients/year) volume surgery. This classification has been used in the literature [6].

### Statistical analysis

All the analyses were performed by using the SAS version 9.3 (SAS Institute, Cary, NC, USA). To analyze differences in the proportion of LBS according to SES groups and their association with demographic characteristics and clinical factors, we performed the chi-square test for categorical variables and the Wilcoxon rank sum test for continuous variables, as appropriate. In addition, a generalized estimating equation (GEE) with a binomial distribution and logit link for dichotomous outcome was applied to adjust for the nesting of patients within physician. The method was used to estimate the impact of SES on the likelihood of undergoing LBS. We further studied the LOS and hospital treatment cost of bariatric surgery between SES groups using a multivariate GEE model with a normal distribution and logit link. Odds ratios (OR) and 95 % confidence intervals (CI) were estimated. The level of statistical significance was set at *P* < 0.05.

## Results

We identified 3678 patients who underwent bariatric procedure between 2004 and 2011 in Taiwan, of whom 3084 cases (83.9 %) were treated by LBS. Basic description of the sample characteristics and LBS rate are shown in Table 1. Patients with high SES (88.1 %) had the highest percentage of undergoing LBS than those with medium (84.6 %) and low SES (80.2 %, *P* < 0.001). The mean age of high SES patients was older (35.7 years) than that of those with medium and low SES (32.7 and 31.8 years, respectively; *P* < 0.001). High SES patients (48.0 %) had the greatest proportion of men, among those with medium SES and low (36.5 % and 40.4 %, *P* < 0.001). Approximately 69.1 % of all the study subjects were living or working in the northern region. However, there was no difference in the patients’ Charlson Comorbidity Index (CCI) between the groups. In terms of hospital and surgeon characteristics, high SES patients who underwent bariatric surgery were more likely to be treated at regional hospitals and by high-volume surgeons compared with other SES groups.

**Table 1:** Characteristics of sample and laparoscopic bariatric surgery (LBS) rate by socioeconomic status (SES) groups in Taiwan

	SES			*P* value
	High (*N* = 745)	Medium (*N* = 1705)	Low (*N* = 1228)	
	*n* (%)	*n* (%)	*n* (%)	
Bariatric procedures				<0.001
LBS	656 (88.1)	1443 (84.6)	985 (80.2)	
OBS	89 (11.9)	262 (15.4)	243 (19.8)	
Patient characteristics				
Age (years), mean (SD)	35.7 (9.2)	32.7 (9.3)	31.8 (9.0)	<0.001
18–29	206 (27.6)	753 (44.2)	596 (48.5)	<0.001
30–39	304 (40.8)	589 (34.5)	395 (32.2)	
40–49	163 (21.9)	242 (14.2)	179 (14.6)	
≥ 50	72 (9.7)	121 (7.1)	58 (4.7)	
Gender				<0.001
Male	358 (48.0)	623 (36.5)	496 (40.4)	
Female	387 (52.0)	1082 (63.5)	732 (59.6)	
Geographic location				<0.001
Northern	578 (77.6)	1140 (66.9)	823 (67.0)	
Central	59 (7.9)	248 (14.5)	216 (17.6)	
Southern	92 (12.3)	281 (16.5)	166 (13.5)	
Eastern	16 (2.2)	36 (2.1)	23 (1.9)	
CCI score				
< 3	679 (91.1)	1578 (92.5)	1109 (90.3)	0.091
≥ 3	66 (8.9)	127 (7.5)	119 (9.7)	
Comorbidity^a^				
Diabetes mellitus	168 (22.6)	309 (18.1)	213 (17.4)	0.011
Dyslipidemia	138 (18.5)	352 (20.7)	247 (20.1)	0.481
Hypertension	248 (33.3)	466 (27.3)	300 (24.4)	<0.001
Obstructive sleep apnea	68 (9.1)	124 (7.3)	81 (6.6)	0.109
Hospital accreditation				0.015
Medical centers	150 (20.1)	371 (21.8)	313 (25.5)	
Regional hospitals	582 (78.1)	1286 (75.4)	881 (71.7)	
District hospitals	13 (1.8)	48 (2.8)	34 (2.8)	
Surgeon age (years), mean (SD)	46.3 (7.1)	46.9 (7.3)	46.6 (7.4)	0.203
Surgeon volume (cases per year)				<0.001
High (≥15)	524 (70.3)	1061 (62.2)	722 (58.8)	
Low (<15)	221 (29.7)	644 (37.8)	506 (41.2)	
Year of operation				0.007
2004	54 (7.2)	122 (7.2)	104 (8.5)	
2005	54 (7.2)	134 (7.9)	114 (9.3)	
2006	38 (5.2)	128 (7.5)	86 (7.0)	
2007	59 (7.9)	197 (11.5)	112 (9.1)	
2008	104 (14.0)	227 (13.3)	145 (11.8)	
2009	91 (12.2)	225 (13.2)	175 (14.2)	
2010	180 (24.2)	330 (19.3)	227 (18.5)	
2011	165 (22.1)	342 (20.1)	265 (21.6)	

^a^A study subject could have more than one comorbidity

The univariate analysis revealed that morbidly obese patients who had high and medium SES were more likely to undergo LBS than those with low SES. However, except for the age, gender, geographic location and CCI score of patients, the hospital accreditation and surgical volume were also significant predictors of undergoing LBS.

After adjusting for patient demographics, hospital and surgeon characteristics, the multivariate analysis revealed that the highest probability of undergoing LBS was noted in patients with high SES (OR = 1.48, 95 % CI 1.13–1.95, *P* = 0.004), followed by those with medium SES (OR = 1.28, 95 % CI 1.04–1.58, *P* = 0.022). In addition, the probability of undergoing LBS was significantly higher among patients who were living or working in the central and southern regions than those who were living or working in the northern region. In addition, after adjustment, patients who underwent LBS were more frequently treated at regional and district hospitals than at medical centers. Surgeons with high-volume practice had higher likelihood of performing LBS than those with low-volume practice (Table 2).

**Table 2:** Univariate and multivariate analyses of likelihood of laparoscopic bariatric surgery (LBS) by socioeconomic status (SES) groups in Taiwan

Variables	Univariate analysis			Multivariate analysis^a^		
	OR	(95 % CI)	*P* value	OR	(95 % CI)	*P* value
SES						
Low	1.00			1.00		
Medium	1.36	(1.12–1.65)	0.002	1.28	(1.04–1.58)	0.022
High	1.82	(1.42–2.33)	<0.001	1.48	(1.13–1.95)	0.004
Patient characteristics						
Age (years)						
18–29	1.00			1.00		
30–39	1.14	(0.90–1.43)	0.281	0.83	(0.65–1.07)	0.157
40–49	0.93	(0.65–1.33)	0.705	0.79	(0.55–1.12)	0.184
≥ 50	0.78	(0.41–1.50)	0.463	0.71	(0.32–1.57)	0.401
Gender						
Male	1.00			1.00		
Female	0.95	(0.74–1.24)	0.727	0.87	(0.66–1.15)	0.332
Geographic location						
Northern	1.00			1.00		
Central	1.19	(0.61–2.34)	0.611	1.97	(1.23–3.16)	0.005
Southern	1.14	(0.56–2.34)	0.714	2.72	(1.42–5.20)	0.002
Eastern	0.42	(0.14–1.21)	0.108	0.83	(0.48–1.45)	0.921
CCI score						
< 3	1.00			1.00		
≥ 3	1.12	(0.72–1.75)	0.601	1.09	(0.72–1.64)	0.683
Hospital accreditation						
Medical centers	1.00			1.00		
Regional hospitals	10.09	(4.05–25.09)	<0.001	4.58	(2.10–9.99)	<0.001
District hospitals	5.15	(2.71–20.53)	<0.001	7.16	(2.07–24.79)	0.002
Surgeon age (years)	1.00	(0.99–1.02)	0.949	0.95	(0.90–1.00)	0.053
Surgeon surgical volume (cases per year)						
Low (<15)	1.00			1.00		
High (≥15)	10.11	(5.24–19.51)	<0.001	8.24	(2.90–23.39)	<0.001
Year of operation						
2004	1.00			1.00		
2005	0.91	(0.46–1.78)	0.774	0.73	(0.39–1.37)	0.326
2006	0.93	(0.16–5.53)	0.938	0.85	(0.32–2.30)	0.755
2007	1.53	(0.23–10.25)	0.66	1.78	(0.67–4.71)	0.247
2008	2.09	(0.27–16.37)	0.483	2.46	(0.82–7.36)	0.109
2009	2.13	(0.31–14.89)	0.445	2.62	(0.69–9.89)	0.156
2010	2.82	(0.45–17.76)	0.269	3.79	(1.17–12.30)	0.026
2011	4.86	(0.76–31.16)	0.096	8.24	(2.59–26.18)	0.001

^a^Adjusted for patient characteristics, hospital accreditation, age and surgical volume of surgeon, and year of operation

Furthermore, the multivariate GEE model, after adjusting for the same covariates, showed that medium SES patients (OR = 0.65, 95 % CI 0.47–0.89, *P* = 0.007) were less likely to experience surgical related complications compared with those with low SES (Table 3). High SES patients had significantly lower LOS (OR = 0.90, 95 % CI 0.82–0.99, *P* = 0.046) and hospital treatment cost (OR = 0.93, 95 % CI 0.87–0.99, *P* = 0.046) than their counterparts.

**Table 3:** Distribution and multivariate analyses of in-hospital outcomes of bariatric surgery by socioeconomic status (SES) groups in Taiwan

	Low (*N* = 1228)	Medium (*N* = 1705)				High (*N* = 745)			
	*n* (%)	*n* (%)	Adjusted OR^a^	(95 % CI)	*P* value	*n* (%)	Adjusted OR^a^	(95 % CI)	*P* value
Surgical complications	30 (2.4)	27 (1.6)	0.65	(0.47–0.89)	0.007	17 (2.2)	1.00	(0.62–1.65)	0.959
LOS (days), median (IQR)	5 (4–8)	5 (4–8)	0.96	(0.89–1.03)	0.339	5 (4–7)	0.90	(0.82–0.99)	0.046
Hospital treatment cost (NT$), median (IQR)	71,749 (57,809–89,787)	70,866 (58,808–84,383)	0.98	(0.92–1.04)	0.477	68,118 (53,163–84,398)	0.93	(0.89–0.99)	0.046

^a^Adjusted for patient characteristics, hospital accreditation, age and surgical volume of surgeon, and year of operation, as compared with patients with low SES

## Discussion

The application of bariatric surgery has been rapidly growing, in part owing to the introduction of minimally invasive techniques and its significant weight loss effect. This analysis of nationally representative data examined patients’ SES in conjunction with the use of LBS for treatment of morbid obesity under the universal healthcare insurance system in Taiwan. We found that morbidly obese patients who had high SES were associated with a high likelihood of undergoing LBS, even after controlling for relevant covariates.

Our findings are similar with data from a study in the United States that reported a higher rate of utilization of the minimally invasive bariatric surgery among higher SES patients and those with private insurance [19]. Most patients treated with minimally invasive approach because of smaller incisions and quicker recovery [2, 3]. In addition, morbidly obese patients who had undergone bariatric surgery typically had multiple obesity-related comorbidities that might have substantially increased their surgical risk [20]. A previous study indicated that patients who undergo open operations are more likely to experience a postoperative complication, especially pulmonary complications, cardiovascular complications, complications during the procedure, sepsis and anastomotic leakage, than those who undergo LBS [21]. Thus, patients with more severe comorbidities may have lower occurrence of undergoing minimally invasive bariatric surgery [1, 7, 19]. The consideration of safety may also explain why patients with higher SES and those with mild severity were more likely to undergo LBS.

Compared to the conventional OBS, patients with a high risk of gastric ulcer, early gastrointestinal bleeding and small bowel obstruction may influence the performance of the LBS [21]. Gastrointestinal bleeding during laparoscopy is quite difficult to control and is also the main reason for conversion to an open surgery. In addition, patients who have had previous abdominal surgery or have complex medical problems such as severe heart and lung diseases may require the open approach [22].

We also found that high SES patients who underwent bariatric surgery had lower LOS and hospital treatment cost. Prior research reported that patients with fewer comorbidities and high volume hospitals and surgeons had better patient outcomes than their counterparts [6, 14]. In addition, the adoption of LBS was also associated with lower LOS when compared open bariatric surgery [1, 3, 7, 23]. This may explain why a decrease in LOS and hospital treatment cost was observed among high SES patients with undergoing bariatric surgery.

The introduction of laparoscopic techniques has revolutionized the field of bariatric surgery during the last decade. However, Taiwan’s National Health Insurance Administration regulated that morbidly obese patients aged 18–55 years and with body mass indexes greater than 40 or 35 kg/m^2^ who had at least one comorbidity are to be indicated for bariatric surgery. This was also recommended by the Asia Pacific Bariatric Surgery Group [24]. Consistent with previous studies [1, 3, 6], the number of LBS procedures performed annually for the treatment of morbid obesity has grown substantially in recent years. In this study, approximately 81.6 % of the patients underwent LBS, despite its technical difficulty and perioperative risks. Previous studies reported that outcomes of LBS improved with the improvement of surgeons’ learning curve for laparoscopic procedures [9]. In addition, surgeons with a high volume of practice with LBS may perceive fewer barriers to performing minimally invasive bariatric surgeries [6, 9]. Hence, the surgical volume and operative experience of the surgeon in laparoscopy-assisted procedures may partially explain the discrepancy in the use of LBS between the study groups.

The present study contributes to the existing literature by examining the effect of socioeconomic differences in the use of LBS in morbidly obese patients who are covered by the universal healthcare insurance. Despite patients’ ability to make informed choice about the surgical approach for obesity treatment, most patients are generally not systematically involved in surgical decisions concerning the advantages and disadvantages of LBS. Therefore, it is important to know how different levels of SES among morbidly obese patients play a role in the use of LBS when selecting surgical candidates. In addition, this study used a large sample of morbidly obese patients in order to achieve a sufficient level of accuracy to detect the relationship between patients’ SES and use of LBS.

Despite showing some improvements in outcomes with laparoscopic bariatric techniques, morbidly obese patients of low SES had substantially lower occurrence of LBS and considerably poorer outcomes than those of high SES. LBS use was associated with patient demographics, timely access to healthcare services based on clinical need and the ability to pay for surgery [13, 19]. Few studies, either administrative or clinical, have examined the cost of LBS in comparison with open operation, as measured by either self-paid charges or actual costs paid by the NHIA. In the Western countries, the admission fee is comparatively more expensive than that in Taiwan [6]. The average surgical cost per patient approximately ranged from NT$ 150,000 for laparoscopic gastroplasty to NT$ 250,000 for laparoscopic gastric bypass surgery or laparoscopic sleeve gastrectomy. However, there is wide variation in charges for bariatric surgery between hospitals that data on actual charges between LBS and OBS has not been reported. In addition, Taiwan’s NHI only covers the fees for hospital stay, operation and anesthesia and medicine (approximately NT$ 20,000), excluding the more expensive instruments and materials for bariatric surgery. Thus, this expensive self-paid cost may lead more low SES people to refuse undergoing LBS, which may be a main cause of our results.

Several limitations of this study should also be noted. First, although the different laparoscopic procedures involved in LBS were broadly classified, this method was clearly defined based on ICD-9-CM procedure codes and previously published literature [18]. In addition, the NHIRD does not allow us to know which patients had converted to bariatric procedures. Second, information on body mass index, dietary or smoking habits and operation time was not available. These factors may also influence the likelihood of undergoing LBS. Nevertheless, we were unable to assess these variables in the NHIRD. Thus, we were unable to conduct more sophisticated analysis including adjustment for such variables due to data limitation. Third, the generalizability of the results may be a concern. We may not be able to generalize these results to patients who have the capacity to pay for bariatric surgery. However, no reasons are apparent for these socioeconomic disparities in access to bariatric surgery under Taiwan’s national health insurance system are apparent and this issue requires further investigation.

## Conclusion

In summary, this study offers evidence that patient’s SES appeared to influence the use of LBS. However, patients’ clinical conditions and surgeons’ practice patterns may also explain the variation in the approaches to LBS. We did note that higher SES was associated with increased odds of undergoing LBS, which may suggest there is the need to improve clinical practice and reduce health disparities in the surgical treatment of morbidly obese patients.

## Abbreviations

CCI: Charlson Comorbidity Index
CI: confidence interval
GEE: generalized estimating equation
ICD-9-CM: International Classification of Diseases, Ninth Revision, Clinical Modification
IQR: interquartile range
LBS: laparoscopic bariatric surgery
LOS: length of hospital stay
NHI: National Health Insurance
NHIA: National Health Insurance Administration
NHIRD: National Health Insurance Research Database
NHRI: National Health Research Institutes
NT$: new Taiwan dollars
OBS: open bariatric surgery
OR: odds ratio
SES: socioeconomic status
